# Hexagonal Close-packed Iron Hydride behind the Conventional Phase Diagram

**DOI:** 10.1038/s41598-019-48817-7

**Published:** 2019-08-23

**Authors:** Akihiko Machida, Hiroyuki Saitoh, Takanori Hattori, Asami Sano-Furukawa, Ken-ichi Funakoshi, Toyoto Sato, Shin-ichi Orimo, Katsutoshi Aoki

**Affiliations:** 1 Quantum Beam Science Research Directorate, National Institutes for Quantum and Radiological Science and Technology, 1-1-1, Kouto, Sayo-cho, Sayo-gun, Hyogo 679-5148 Japan; 2 J-PARC Center, Japan Atomic Energy Agency, Tokai, Naka, Ibaraki, 319-1195 Japan; 3 Neutron Science and Technology Center, Comprehensive Research Organization for Science and Society, Shirakata, Tokai, Naka, Ibaraki, 319-1106 Japan; 4 Institute for Materials Research, Tohoku University, 2-1-1 Katahira, Aoba-ku, Sendai 980-8577 Japan; 5 WPI-Advanced Institute for Materials Research (AIMR), Tohoku University, 2-1-1, Katahira, Aoba-ku, Sendai 980-8577 Japan; 6 Graduate School of Science, The University of Tokyo, 7-3-1 Hongo, Bunkyo-ku, Tokyo 113-0033 Japan

**Keywords:** Chemical physics, Condensed-matter physics

## Abstract

Hexagonal close-packed iron hydride, hcp FeH_*x*_, is absent from the conventional phase diagram of the Fe–H system, although hcp metallic Fe exists stably over extensive temperature (*T*) and pressure (*P*) conditions, including those corresponding to the Earth’s inner core. *In situ* X-ray and neutron diffraction measurements at temperatures ranging from 298 to 1073 K and H pressures ranging from 4 to 7 GPa revealed that the hcp hydride was formed for FeH_*x*_ compositions when *x* < 0.6. Hydrogen atoms occupied the octahedral interstitial sites of the host metal lattice both partially and randomly. The hcp hydride exhibited a H-induced volume expansion of 2.48(5) Å^3^/H-atom, which was larger than that of the face-centered cubic (fcc) hydride. The hcp hydride showed an increase in *x* with *T*, whereas the fcc hydride showed a corresponding decrease. The present study provides guidance for further investigations of the Fe–H system over an extensive *x–T–P* region.

## Introduction

Transition metals react with hydrogen to form hydrides, MH_*x*_, at hydrogen pressures of several gigapascals (GPa; hereafter, the hydrogen pressure is referred to simply as “pressure”)^1^. Hydrogen molecules dissociate to hydrogen (H) atoms on the metal surface, where the H atoms dissolve into the bulk to partially or fully occupy the interstitial sites of the metal lattice. The interstitial H atoms expand the volume of the metal lattice by 10–20% for a MH_*x*_ composition of *x* = 1^1^. The H composition *x* varies as a function of the temperature (*T*) and pressure (*P*). Accordingly, the volume of hydride (*V*) varies via a hydrogen-induced volume expansion. In addition to *V*, *x* is an essential variable for describing the bulk state of a hydride. Hydrogen compositions for recovered specimens have been measured by neutron diffraction or hot extraction of H_2_ gas at ambient pressure^2^. *In situ* neutron diffraction was recently used to investigate the structure of iron deuteride under high *T–P* conditions, and the D composition was successfully determined^3^.

**Figure 1:**
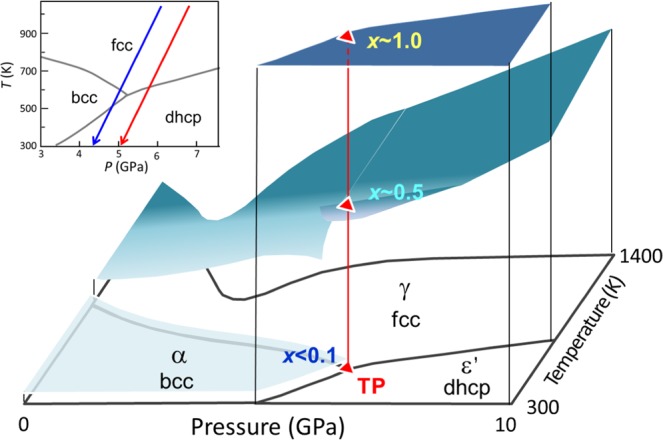
Schematic of the *x–T–P* diagram of the Fe–H system: the inset is a projection onto the *T–P* plane showing *T–P* paths used in X-ray (red arrow) and neutron (blue arrow) diffraction measurements.

Iron hydride (FeH_*x*_) has been intensively studied for half a century as a prototype of transition-metal hydrides^2–13^ and an endmember of the constituents of the Earth’s core^5,14–24^. Figure 1, plotted from previously reported data^2–10^, shows a schematic of the *x–T–P* diagram of the Fe–H system at temperatures from 300 to 1400 K and pressures ranging from 0 to 10 GPa ^2–10^. Three phases exist: a low-pressure α phase with a body-centered cubic (bcc) structure, wherein Fe atoms occupy the vertexes of the bcc lattice; a high-temperature γ phase with a face-centered cubic (fcc) structure and a high-pressure εʹ phase with a double hexagonal close-packed (dhcp) structure. The bcc and fcc phases are a solid solution of hydrogen for *x* <1.0, whereas the dhcp phase is a monohydride for *x* = 1 over almost the entire stable *T–P* region. The triple point is located at approximately 520 K and 5 GPa^2,4,9,10^, where the bcc, dhcp, and fcc phases have approximate H compositions of 0.1, 1.0, and 0.5, respectively^5,7–10^. The phase stability for iron hydride in equilibrium with fluid hydrogen has been investigated, where the composition *x* was uniquely fixed to the highest value at a given *T–P* condition for each hydride phase. A different hydride should form for *x* values below the equilibrium *x* surface. The hcp hydride is absent in the conventional phase diagram although the hcp phase of metallic iron is stable at extensive *T–P* conditions up to those corresponding to the Earth’s inner core^25^.

We performed structural investigations on the Fe–H system to explore the formation of the hcp hydride at *x–T–P* conditions below the equilibrium *x* surface. *In situ* X-ray diffraction measurements revealed that the hcp hydride appeared at ~800 K while the fcc hydride was cooled from ~1000 K at ~7 GPa under conditions where there was no coexisting fluid H_2_ (red arrow in the inset of Fig. 1). The hcp hydride subsequently decomposed into dhcp FeH and bcc Fe at ~430 K upon further cooling. The crystal structure including the site occupancy of deuterium (D) atoms was investigated via *in situ* neutron diffraction measurements of the hcp deuteride, which was prepared by cooling the fcc deuteride (blue arrow in the inset). In this communication, we present *x*–*T–P* conditions for the formation of the hcp hydride and the variation of *x* with the fcc–hcp–dhcp structural transitions. Hydrogen-induced volume expansion and the *x–T* relation, which are essential properties for characterizing metal hydrides, are derived from the structural data and compared with those of the fcc hydride.

## Results

### X-ray diffraction measurements

The X-ray diffraction profiles were collected while cooling fcc FeH_*x*_ with *x* ≈ 0.6 from 1073 K to 298 K at an initial pressure of ~7 GPa. In this experiment, the amount of aluminum trihydride (AlH_3_) pellets that was used as an internal H source was reduced to a H/Fe molar ratio of ~0.6 to prevent transformation from fcc FeH_*x*_ to the monohydride dhcp FeH with additional H absorption. The temperature was continuously decreased at 10 K/min, and the pressure was reduced from 7.1 to 5.0 GPa because of the thermal contraction of the reaction cell. Time-resolved X-ray diffraction profiles were collected with an exposure time of 20 s/profile using the energy dispersion method^26^.

**Figure 2:**
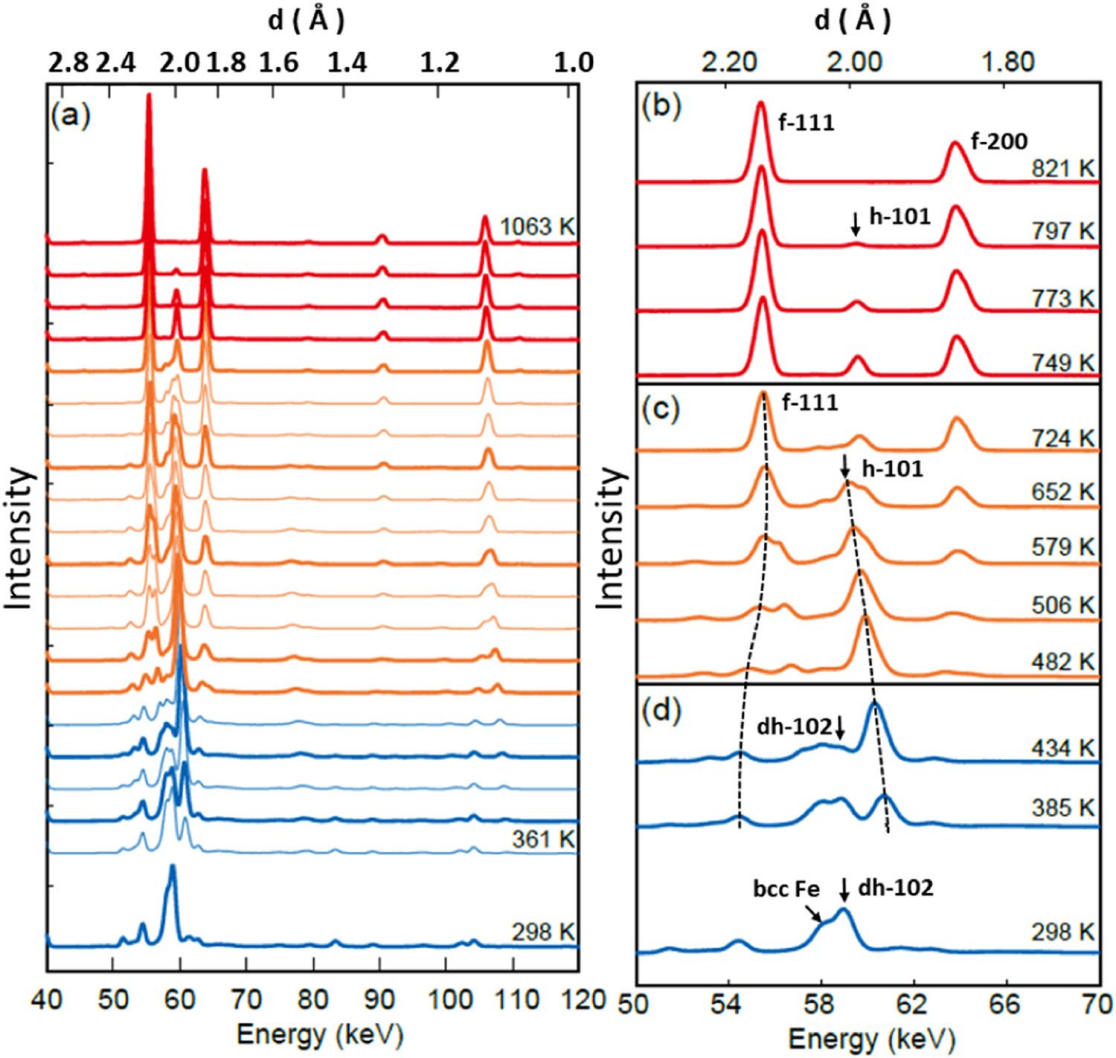
X-ray diffraction profiles collected for iron hydride upon cooling, showing (**a**) overall evolution and (**b**) fcc-dominant, (**c**) hcp-dominant, and (**d**) dhcp-dominant regions; reflection indices are assigned to the major peaks of each structure, where the shifts of the fcc 111 and hcp 101 peaks are indicated by broken lines to guide the eye.

Figure 2(a) Shows the evolution of the diffraction profile with decreasing temperature. The observed profiles are divided into approximately three regions for ease of explanation: fcc-dominant (Fig. 2(b)), hcp-dominant (Fig. 2(c)), and dhcp-dominant regions (Fig. 2(d)).

When the temperature decreased to ~800 K, only one peak appeared (~60 keV, *d* ≈ 2.0 Å) in addition to those of the fcc hydride, as shown in Fig. 2(b). The peak intensity increased with further decrease in the temperature to ~750 K, whereas the peak intensity of the fcc peaks remained unchanged. Other new peaks appeared at ~650 K. These peaks were assigned as hcp lattice reflections, indicating a transformation from the fcc to the hcp structure. The most intense 101 peak (denoted by the arrow in Fig. 2(c)), which appeared on the low-energy side of the preceding 101 peak, was shifted to a higher energy with decreasing temperature, thereby merging with the energy level of the 101 peak in Fig. 2(b). We assigned the preceding peak at temperatures of 800–750 K as a hcp 101 peak from “precipitated hcp hydride” and the peaks appearing at ~650 K were assigned to “transformed hcp hydride.”

The fcc–hcp structural transformation proceeded until ~480 K (Fig. 2(c)). The fcc 111 peak was shifted to a lower energy because of volume expansion with H absorption; by contrast, the hcp 101 peak was shifted to a higher energy because of volume contraction with H desorption. The hcp hydride eventually decomposed into dhcp FeH_*x*_ (*x* ≈ 1.0) and bcc Fe (Fig. 2(d)) at ~430 K. The diffraction profiles showed peaks from the fcc and hcp hydrides at temperatures below ~650 and ~430 K, respectively. A small amount of the fcc and hcp hydrides remained in a metastable state, probably because of slow transformation kinetics at lower temperatures. The diffraction peaks continued to shift to lower or higher energies in the metastable temperature ranges, and the corresponding peak positions were used to calculate the lattice constants or atomic volumes of Fe, as described in the following paragraphs.

### Neutron diffraction measurements

We performed neutron diffraction measurements with the same reaction cell as that used in the X-ray diffraction experiments, except that the AlH_3_ pellet was replaced with an AlD_3_ pellet^3,27,28^. An excess amount of AlD_3_ with a Fe/D molar ratio of ~1.5 was charged into the cell to completely deuterize a bulk Fe specimen, 3.0 mm in diameter and 2.5 mm in thickness, within a short time. However, deuterization of the Fe disc took 90 min even at temperatures as high as 1073 K. By contrast, only a few minutes were required for the hydrogenation of Fe flakes, which were mixed with the BN powder that was used in the X-ray diffraction experiments. This result was expected because the surface areas for the Fe disc and flakes differed by orders of magnitude. After the formation of the fcc deuteride at 1073 K and 6.0 GPa was confirmed by neutron diffraction, the temperature was rapidly decreased to 673 K to prevent the fcc deuteride from achieving an equilibrium D composition; the hcp deuteride was thus prepared. The temperature was further decreased to 573 K and finally to 300 K. A neutron diffraction profile was collected at each temperature with a few hours of integration time. The pressure decreased from 6.0 to 4.2 GPa upon cooling to 300 K.

**Figure 3:**
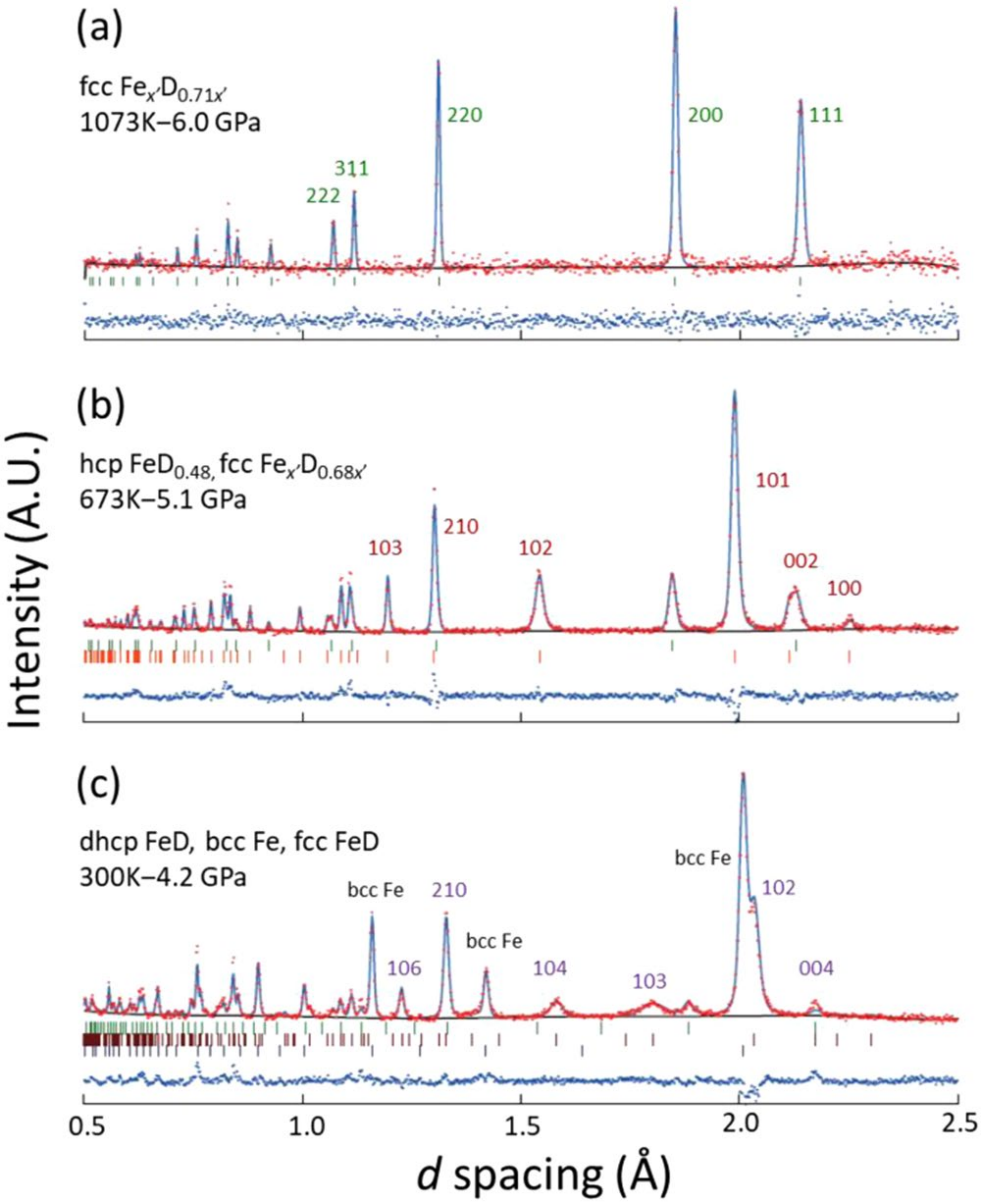
Experimental and simulated neutron diffraction profiles of iron deuteride at (**a**)1073 K and 6.0 GPa, (**b**) 673 K and 5.1 GPa, and (**c**) 300 K and 4.2 GPa: blue lines indicate differences between the experimental (red dots) and simulated (blue curves) profiles; reflection indices are assigned to the major peaks of the dominant structures (**a**) fcc, (**b**) hcp, and (**c**) dhcp; tick marks indicate the positions of allowed Bragg peaks for each structure.

Figure 3 shows the neutron diffraction profiles that were recorded at 1073 K and 6.0 GPa (a), 673 K and 5.1 GPa (b), and 300 K and 4.2 GPa (c). The corresponding simulated and experimental profiles, as fitted by Rietveld refinement^29^, are shown. It should be noted that the Fe and D compositions of the fcc deuteride are denoted by *x*ʹ in the panels (a) and (b). The site occupancy of the Fe atoms in the fcc lattice deviated slightly from unity to *x*ʹ < 1.0 because of the formation of vacancies at the Fe sites^9,10,30^. The Rietveld refinement only provides the ratio of the site occupancies between Fe and D atoms; hence, the Fe composition is described by *x*ʹ.

The diffraction peaks at 673 K showed that the hcp structure was the dominant component. Rietveld refinement using a hcp model structure with D atoms randomly occupying the interstitial sites yielded site occupations of 0.48(1) (hereafter, the numbers in parentheses denote the experimental error) and 0.0 for the octahedral and tetrahedral sites, respectively, and a deuterium composition of *x* = 0.48(1). A very similar diffraction profile was observed for the hcp deuteride at 573 K and 4.8 GPa, yielding *x* = 0.48(1). In the 300-K profile, the dominant diffraction peaks originated from dhcp FeD and bcc Fe; the hcp deuteride decomposed, as observed in the X-ray diffraction experiments. For dhcp FeD, we used a stacking fault model that was presented in the early neutron diffraction study^8^. Because bcc Fe and dhcp Fe(H/D) are ferromagnetic^31,32^, each diffraction peak contains a magnetic scattering component in addition to a nuclear one. The magnetic moment was optimized to 2.1 (1.1) in Bohr magnetons (μ_B_) for bcc Fe. No magnetic contribution considered for dhcp FeD because the peak intensity was too low for the magnetic structure to be refined. The structural parameters that were optimized by Rietveld refinement are summarized in Table 1.

**Table 1:** Positional parameters (*x*, *y*, *z*) and site occupancies for iron deuterides; *X*mass: mass fraction, Z: number of formula units per unit cell, B: atomic displacement parameter.

*T*, *P*, Reliable factors	Phase	Atom	Site	x	y	z	B(Å^2^)	Occupancy
1073 K, 6.0 GPa *R*_wp_ = 25.4%, *χ*^2^ = 0.979	fcc-Fe_*x*′_D_0.71*x*′_, *X*mass = 1.0 Fm-3m, *Z* = 4 a = 3.70298(19) Å	Fe D D	4a 4b 8c	0 1/2 1/4	0 1/2 1/4	0 1/2 1/4	1.2 3.4	*x´* 0.54(2)*x*′ 0.08(1)*x'*
673 K, 5.1 GPa *R*_wp_ = 12.4%, *χ*^2^ = 2.46	hcp-FeD_0.48_, *X*mass = 0.74 P6/mmc, Z = 4 a = 2.60047(10) Å, c = 4.2280(4) Å prref. orient. (001) = 1.08	Fe D	2c 2a	1/3 0	2/3 0	1/4 0	0.74 2.2	1.0 0.48(1)
	fcc-Fe_*x*′_D_0.68*x*′_, *X*mass = 0.26 Fm-3m, Z = 4 a = 3.6901(3) Å	Fe D D	4a 4b 8c	0 1/2 1/4	0 1/2 1/4	0 1/2 1/4	0.74 2.2	*x*′ 0.51(2)*x*′ 0.08(1)*x'*
573 K, 4.8 GPa *R*_wp_ = 13.8%, *χ*^2^ = 2.01	hcp-FeD_0.48_, *X*mass = 0.80 P6/mmc, Z = 4 a = 2.59662(8) Å, c = 4.2155(3) Å prref. orient. (001) = 1.09	Fe D	2c 2a	1/3 0	2/3 0	1/4 0	0.63 1.8	1.0 0.48(1)
	fcc-Fe_*x*′_D_66*x*′_, *X*mass = 0.20 Fm-3m, Z = 4 a = 3.6939(3) Å	Fe D	4a 4b 8c	0 1/2 1/4	0 1/2 1/4	0 1/2 1/4	0.63 1.8	*x*′ 0.51(2)*x*′ 0.07(1)*x'*
300 K, 4.2 GPa *R*_wp_ = 14.4%, *χ*^2^ = 2.71	dhcp-FeD, *X*mass = 0.53 P6/mmc, *M* = 4 a = 2.65605(14) Å, c = 8.6950(15) Å prref. orient. (001) = 1.61	Fe Fe D Fe D	2a 2c 4f 2d 4f	0 1/3 1/3 1/3 1/3	0 2/3 2/3 2/3 2/3	0 1/4 0.8805(9) 3/4	0.29(6) 0.29(6) 0.90(9) 0.29(6) 0.90(9)	1.0 0.79(1) 0.79(1) 0.21(1) 0.21(1)
	bcc-Fe, *X*mass = 0.45 Im-3m, Z = 2 a = 2.83873(10) Å	Fe	2a	0	0	0	0.29(6)	1.0
	fcc-FeD, *X*mass = 0.02 Fm-3m, Z = 4 a = 3.7642(10) Å	Fe D	4a 4b 8c	0 1/2 1/4	0 1/2 1/4	0 1/2 1/4	0.29(6) 0.90(9)	1.0 1.0 0.0

### *V–T* relation

**Figure 4:**
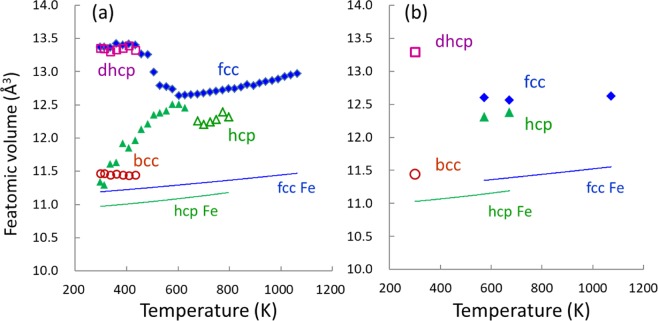
(**a**) The *v*_Fe_–*T* relations of fcc (blue solid diamonds), dhcp (purple open squares), and hcp (green solid triangles) hydrides and bcc Fe (brown open circles) obtained by X-ray diffraction, where the estimated atomic volumes for the precipitated hcp hydride are represented by green open triangles; (**b**) estimated atomic volumes determined by neutron diffraction, where the symbols have the same meaning as in the left panel; in both (**a**,**b**) panels, the calculated *v*_Fe_–*T* relations for hcp Fe and fcc Fe are represented by green and blue lines, respectively.

The atomic volume of Fe, *v*_Fe_, which is calculated by dividing the unit cell volume of iron hydride by the number of Fe atoms contained in the cell, was obtained for each hydride from its X-ray and neutron diffraction data. The atomic volume was plotted as a function of temperature in the 298–1073 K range in Fig. 4. The atomic volumes of the fcc and hcp Fe metals, which were calculated using their equations of state^33,34^, are also plotted as references. For the precipitated hcp hydride, only the 101 peak was observed at temperatures in the 730–800 K range (Fig. 2(b)). The atomic volume for the precipitated hcp hydride was estimated from the measured *d* values of the 101 peak and the axial ratio of *c/a* = 1.600 that was obtained for the transformed hcp hydride. The atomic volumes were also calculated for the fcc and hcp hydrides that remained as metastable states below the transformation and decomposition temperatures, respectively.

The *v*_Fe_–*T* relations in Fig. 4a were used to derive the deuterium-induced volume *v*_D_ = Δ*v*_Fe_/*x*^1^. Here, the excess amount of *v*_D_. that arises from the volume expansion of the metal lattice owing to the dissolution of H/D atoms can be calculated using Δ*v*_Fe_ = *v*_Fe_ (hydride) − *v*_Fe_ (reference metal). The value of *v*_D_ for the hcp deuteride at 673 K and 5.1 GPa was found to be 2.51 (5) Å^3^/D atom using Δ*v*_Fe_ = 1.191 Å^3^, which was calculated from the *v*_Fe_ (hcp FeD_*x*_) and the calculated *v*_Fe_ (hcp Fe, which is plotted in Fig. 4b), and *x* = 0.48(1). Using alternative data for Δ*v*_Fe_ = 1.166 Å^3^ and *x* = 0.48(1) at 573 K and 4.8 GPa, we obtained *v*_D_ = 2.45(4) Å^3^/D atom. We took an averaged value of 2.48(5) Å^3^/D atom for the *v*_D_ of hcp deuteride. For dhcp FeD, a 2.42 (4) Å^3^/D atom was obtained using the structural data that are listed in Table 1, where the hcp Fe volume was used as the reference volume. The volume of ferromagnetic dhcp deuteride contains an unknown contribution from magnetic volume expansion; hence, the calculated value is an upper limit on *v*_D_. The volume data for fcc FeD_*x*_ that were obtained by the neutron diffraction should be regarded with caution because the lattice volume for this deuteride is substantially reduced owing to vacancy formation in the metal lattice^9,10,30^. Hence, we used the *v*_D_ value of 2.21(4) Å^3^/D atom, as has been previously reported for vacancy-free fcc FeD_*x*_^3^. The *v*_D_ of the hcp hydride is the largest volume among those for iron hydrides.

### *x*–*T* relation

**Figure 5:**
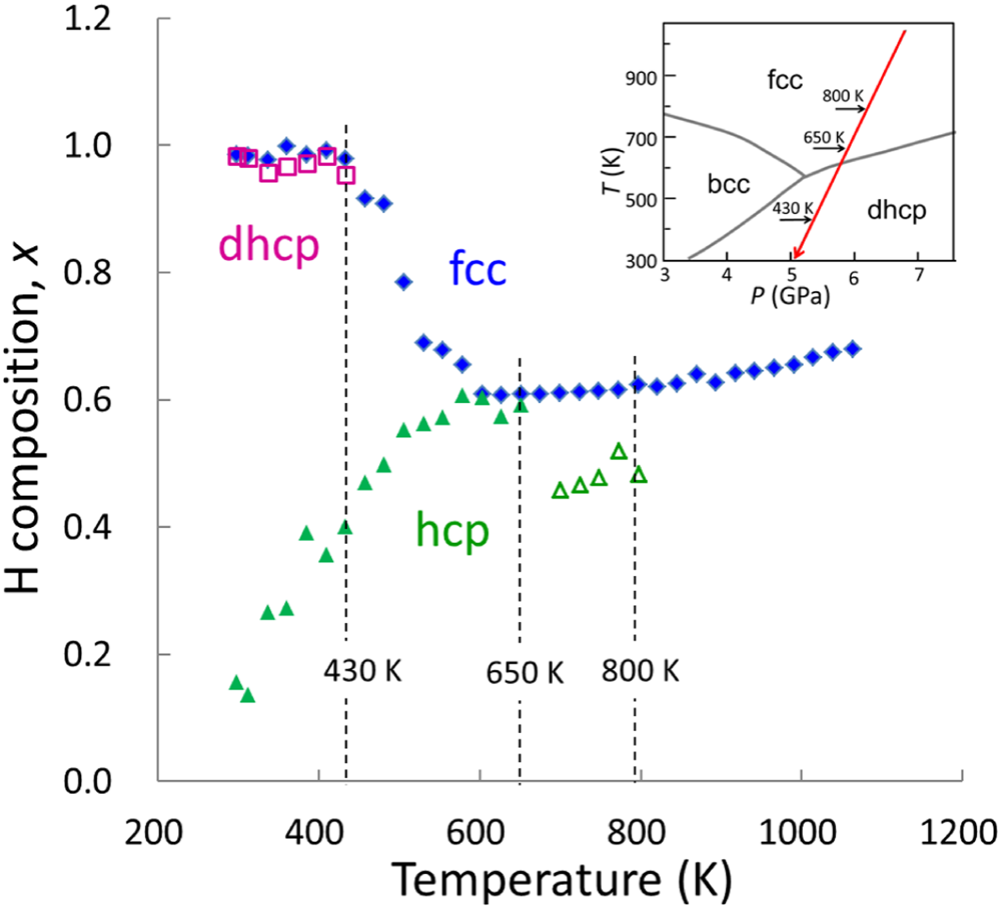
The *x*–*T* relations are shown for fcc (blue solid diamonds), hcp (green solid triangles), and dhcp (purple open squares) hydrides. The estimated compositions for the precipitated hcp hydride are represented by green open triangles. Vertical broken lines show the approximate precipitation, transformation, and decomposition temperatures. The inset shows the cooling *T*–*P* path.

The expanded volume, Δ*v*_Fe_, for the fcc and hcp hydrides that was measured over a temperature range of 298–1073 K by X-ray diffraction was converted to H compositions using a proportionality relation, *x* = Δ*v*_Fe_/*v*_H_, in which *v*_H_ = *v*_D_ is assumed. Figure 5 shows the *x*–*T* relations of the fcc and hcp hydrides that were calculated using *v*_H_ = 2.21 and 2.48 Å^3^/H atom, respectively. The *x* value of the fcc hydride decreased slightly from 0.68 at 1073 K to 0.66 at 600 K, before increasing towards a saturated value of 1.0; the fcc monohydride and the dhcp monohydride formed at temperatures below ~430 K. The value of *x* ≈ 0.5 was obtained for the precipitated hcp hydride in the 700 to 800 K range, whereas the transformed hcp hydride showed a monotonic decrease in *x* with decreasing temperature below 650 K. Despite the very similar values of *x* ≈ 0.6 for the hcp and fcc hydrides at ~600 K, opposing trends were observed for the variation in the H compositions with the temperature below 600 K.

## Discussion

Iron hydride/deuteride with an hcp metal lattice was formed by the transformation from the fcc hydride/deuteride. In the early studies^6,8^, the hcp hydride/deuteride formed as an intermediate metastable state during the hydrogenation/deuterization of bcc Fe to dhcp Fe(H/D). The “transformed” hcp deuteride has the same crystal structure as that of the “intermediate” hcp deuteride as shown by the structural parameters presented in Table I and Table 3 of ref.^8^. The D atoms occupy the octahedral interstitial sites of the host metal lattice both partially and randomly; The present study provided a D composition of 0.48 for hcp deuteride at 673 K and 5.1 GPa, and at 573 K and 4.8 GPa. This value was slightly higher than 0.42 reported for hcp deuteride prepared at 623 K and 9.2 GPa^8^. The tetrahedral site occupation, reported for fcc FeD_*x*_^3^, or the formation of layered octahedral superstructures, reported for hcp TcH_*x*_ and MnD_*x*_ at *x* = ~1/2^35^ was not observed.

The hcp iron hydride, in both its stable and metastable states, formed for *x* < 0.6 at pressures from 4 to 6 GPa (Table 1 and Fig. 5). This hcp hydride lies under the equilibrium *x*-surface in the phase diagram that is shown in Fig. 1. Controlling of the H composition plays a key role in the formation of hcp hydride. In the neutron diffraction measurements, the bulk fcc deuteride transformed to the hcp deuteride through the nonequilibrium state formed due to the relatively low diffusion rate of D atoms at the measured temperatures. In the X-ray diffraction measurements, the powder fcc hydride transformed to the hcp hydride under the condition of insufficient hydrogen supply. Both of the transformations occurred near the stable *T–P* region of dhcp phase; the hcp hydride, instead of the dhcp monohydride, was preferentially precipitated. These results suggest the formation of hcp hydride over a wide *T–P* region under controlled H composition. The most recent theoretical calculations have shown that the hcp hydride becomes more stable than the dhcp hydride when *x* <~0.5 at extensively high *T–P* conditions^36^. Further phase studies of the Fe–H system in extended *x–T–P* conditions are required to clarify the structural stability of the hcp hydride in terms of the H composition.

**Figure 6:**
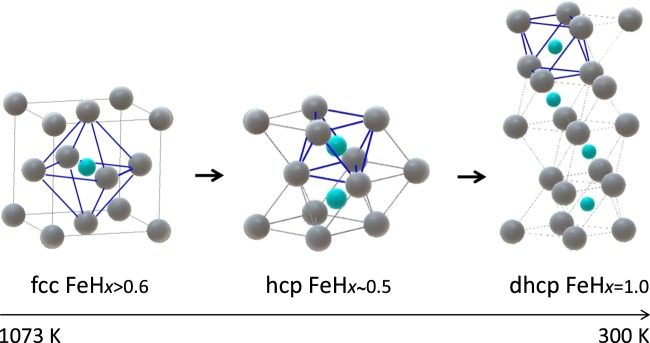
Crystal structures of fcc, hcp, and dhcp iron hydrides: grey and blue spheres represent Fe and H atoms, respectively; dissolved H atoms occupy the octahedral interstitial sites partially in the fcc and hcp hydrides and fully in the dhcp hydride.

The crystal structures of iron hydride that appeared sequentially in the cooling experiments are drawn in Fig. 6. The fcc, hcp, and dhcp structures can transform into each other by sliding metal planes and tuning the H composition. At the fcc–hcp transformation temperature of ~650 K, the H composition of both hydrides is *x* = 0.6; hence, the fcc structure can transform to the hcp structure by simply altering the stacking sequence of the metal planes from ABCABC∙∙∙ to ABAB∙∙∙ along the body diagonal axis of the cubic lattice. For decomposition at ~430 K, the dhcp structure can form by altering the sequence from ABAB∙∙∙ to ABABACAC∙∙∙ along the *c* axis of the hcp lattice and filling all of the octahedral sites with H atoms.

Although each of the metal lattices has one octahedral site per Fe atom available for H-atom accommodation, the spatial arrangements of these lattices are quite different. The octahedra consisting of Fe atoms at the corners are connected by corner sharing in the fcc lattice but by face-sharing in the hcp lattice (Fig. 6). The dhcp lattice consists of a mixture of two configurations, as seen in its sequence ABABACAC∙∙∙. The face-sharing configuration substantially shortens the first-neighbor distance between the H atoms. For the coexisting state at 673 K and 5.06 GPa, the fcc lattice constant of *a* = 3.6901(3) Å and the hcp lattice constants of *a* = 2.60047(10) Å and *c* = 4.2280(4) Å were obtained (Table 1). These values provide first-neighbor distances of 2.609 Å and 2.114 Å for the fcc and hcp structures, respectively. The latter distance of 2.114 Å is very close to the critical distance of 2.1 Å, below which dissolved H atoms in metals cannot approach each other owing to interatomic repulsion forces^37^. Dissolved H atoms can preferentially occupy second-neighbor octahedral sites to avoid violating the 2.1-Å rule in the half-filled hcp lattice but not in the hcp monohydride. The 2.1-Å rule is a possible factor in the stabilization of the hcp structure for *x* < 0.6.

The hcp and fcc solid solutions exhibited opposing variations in *x* with the temperature. The two-step variation of the fcc hydride was interpreted in terms of a miscibility gap; solid solutions with high and low H compositions can coexist below a critical *T–P* point, at which the H solubility gap vanishes. For the fcc hydride, the miscibility gap was confirmed experimentally^10,13^ and the critical pressure was located at 4.0*–*4.5 GPa at a critical composition of *x* ≈ 0.4^13^. The measured pressure range of 5.0*–*7.1 GPa was higher than the critical pressure; hence, the H composition of the fcc hydride increased along the high-composition boundary of the miscibility gap with decreasing temperature below ~600 K, as shown in Fig. 5. For the hcp hydride, a miscibility gap has been theoretically predicted^38^, but has not been experimentally confirmed. The observed monotonic decrease in the *x–T* curve for the hcp hydride that is shown in Fig. 5 implies that the critical pressure was above ~6 GPa. Consequently, the H composition decreased with decreasing temperature along the low-composition boundary of the miscibility gap.

## Methods

### X-ray diffraction

The starting material was reagent-grade pure iron flakes (purity: 99.9%) with a lateral particle size <100 μm and a thickness <20 μm. The flakes were mixed with BN powder (purity: 99% and grain size: >10 μm) at a volume ratio of 2:3 and compacted into a disc that was 0.5 mm in diameter and 0.2 mm in height. The sample disc was loaded along with a compacted AlH_3_ disc, which served as an internal H source, into a sleeve made of pyrolytic BN. This sleeve was placed into a NaCl capsule that was surrounded by a cylindrical graphite heater. The cell assembly was performed in the air. High pressures and temperatures were generated using a cubic-type multi-anvil press. The internal H source decomposed into fluid H_2_ and Al metal upon heating above 800 K. The fluid H_2_ reacted with the Fe specimen to form FeH_*x*_ in the NaCl capsule. The temperature was monitored using Pt/Pt*–*13%Rh thermocouples with an uncertainty of less than 20 K. *In situ* X-ray diffraction measurements were conducted using synchrotron radiation at the BL14B1 beamline of SPring-8. Details of the high-pressure generation, the hydrogenation cell, and the *in situ* synchrotron-radiation X-ray diffraction technique are described elsewhere^26^.

### Neutron diffraction

The cell assembly for the high-pressure neutron diffraction measurements was essentially the same as that used for X-ray diffraction. A compacted Fe disc, 3 mm in diameter and 2.5 mm in height, was prepared by pressing Fe flakes in a piston-cylinder-type mold. The Fe specimen was placed at the center of a NaCl capsule (5.5 mm in diameter and 8 mm in height) and AlD_3_ (isotopic purity: 96 atom% D) pellets, which served as an internal D source, was placed above and below the Fe specimen. The NaCl capsule was inserted into a cylindrical graphite heater and embedded in a pressure-transmitting medium made of MgO (17-mm edge cube). The cell assembly was performed in the air. *In situ* neutron diffraction measurements were conducted using the pulsed neutron source at the BL 11 (PLANET) beamline of J-PARC^27^. The collected diffraction profiles were refined using Z-Rietveld software (version 0.9.42.2)^29^. In the refinement, H atoms that were included as an impurity at four atom% were assumed to randomly occupy the D atom sites. For simplicity, the site occupancies of the H atoms and the H composition are notated as gD and x, respectively. The cell assembly and the high-pressure apparatus that was used for the neutron diffraction experiments are described in detail elsewhere^3^.

## Data Availability

All data supporting the findings of this study are available within the paper and Methods. The crystallographic data are available from the corresponding authors upon request.
